# Circadian rhythm in mycotoxin-induced immunotoxicity: an emerging regulatory axis?

**DOI:** 10.3389/fphar.2025.1700863

**Published:** 2025-10-23

**Authors:** Li You, Eugenie Nepovimova, Klara Sklenarikova, Qinghua Wu, Kamil Kuca

**Affiliations:** ^1^ College of Physical Education and Health, Chongqing College of International Business and Economics, Chongqing, China; ^2^ College of Life Science, Yangtze University, Jingzhou, China; ^3^ Department of Chemistry, Faculty of Science, University of Hradec Králové, Hradec Králové, Czechia; ^4^ Center of Advanced Innovation Technologies, VSB-Technical University of Ostrava, Ostrava-Poruba, Czechia; ^5^ Faculty of Informatics and Management, University of Hradec Kralove, Hradec Kralove, Czechia; ^6^ Biomedical Research Center, University Hospital Hradec Kralove, Hradec Kralove, Czechia

**Keywords:** mycotoxins, circadian rhythm, *BMAL1*, oxidative stress, immunosuppression

## Abstract

Mycotoxins, toxic secondary metabolites produced by fungi, pose a substantial worldwide health risk due to their widespread contamination of food commodities. Their toxicological effects include organ dysfunction, oxidative stress, and suppression of immune function. Emerging data indicate that circadian rhythm disruption is a critical but underrecognized mechanism contributing to mycotoxin-induced toxicity. This review summarizes current evidence showing that mycotoxins directly interfere with molecular circadian rhythm regulators. Specifically, deoxynivalenol markedly downregulates the expression of *BMAL1*, *CLOCK*, and *CRY1/2* in hepatic cells. Similarly, zearalenone perturbs the temporal expression of *BMAL1*, *PER2*, and *NR1D1* in testicular tissue, impairing testosterone biosynthesis. Furthermore, circadian rhythm disruption triggered by mycotoxins may initiate downstream pathological responses, including enhanced ROS generation and immune dysfunction through *BMAL1*-dependent regulation of *PD*-*L1* expression. Importantly, a reciprocal feedback loop appears to exist wherein oxidative stress intensifies circadian rhythm disruption, which in turn promotes ROS accumulation and further immune impairment. These insights establish circadian rhythm disruption as a central mediator of mycotoxin-related toxicity and highlight *BMAL1* as a potential therapeutic target. Nonetheless, experimental validation remains limited, and further mechanistic studies are required. We propose that circadian rhythm disruption may serve as an integrative node within the mycotoxin toxicity pathway, linking oxidative imbalance to immunosuppressive outcomes.

## 1 Introduction

Mycotoxins are secondary metabolites produced by toxigenic fungi, through specific biosynthetic pathways (Tian et al., 2022; Wang et al., 2022). In wheat, the most prevalent mycotoxins are deoxynivalenol (DON) and zearalenone (ZEA). They are type B trichothecenes and estrogenic mycotoxins, respectively, and are primarily synthesized by *Fusarium*. Other significant mycotoxins include aflatoxin B1 (AFB1), derived from *Aspergillus flavus*; T-2 toxin, from *Fusarium genus*; and ochratoxin A (OTA) produced by *Aspergillus and Penicillium* (Sun et al., 2022; Wang et al., 2022). These toxins accumulate in animals and humans through the food chain, posing serious health risks such as hepatotoxic, nephrotoxic, genotoxic, and immunosuppressive effects, which can lead to carcinogenesis or death (Kościelecka et al., 2023; Shekhar et al., 2025). The molecular mechanisms underlying their toxicity are increasingly being elucidated. For instance, AFB1 induces liver damage via the Toll-like receptor 4 (TLR4)/receptor-interacting protein kinase 1/3 (RIPK1/3) signaling pathway (Li et al., 2020; Li et al., 2022) and Kelch-like ECH-associated protein 1 (Keap1)/nuclear factor erythroid 2-related factor 2 (NRF2) pathway (Li et al., 2020; Zhao et al., 2021a) OTA contributes to glomerular injury by activating the extracellular signal-regulated kinase (ERK)/nuclear factor-κB pathway (Darbuka et al., 2021; Le et al., 2020). It also promotes inflammation via the gut-liver axis by increasing *Bacteroides* abundance in the intestine, enhancing lipopolysaccharide release and subsequent activation of the TLR4-myeloid differentiation factor 88 cascade (Ruan et al., 2019; Wang et al., 2019). DON compromises intestinal epithelial integrity by altering villus structure and disrupting mucosal barrier function (Li et al., 2021; Moldal et al., 2018). Mechanistically, DON activates the ERK and p38/MAPK signaling pathways, leading to suppressed Claudin-4 expression and lysosome-mediated degradation of Occludin and ZO-1 (Li et al., 2021; Wang et al., 2021). These alterations result in villus structural damage and increased intestinal permeability. While these established pathways effectively explain the direct organ damage caused by mycotoxins, they may not fully account for the systemic and progressive nature of their toxicity. Consequently, research has expanded to explore more fundamental regulatory systems.

Beyond these established pathways, emerging evidence suggests that mycotoxins may also exert toxicity by disrupting the endogenous circadian timing system, a fundamental regulator of physiology (Yang et al., 2023; Zhao et al., 2021b). On the one hand, the fungal biological clock precisely regulates toxin synthesis (such as the rhythmic expression of the OTA polyketide synthase gene) and the circadian rhythm of host infection (such as *Fusarium oxysporum* coordinating the virulence sequence through the transcription factors *FoZafA*/*FoCzf1*) (Lu et al., 2025; Schmidt-Heydt et al., 2010). On the other hand, mycotoxins (such as DON and ZEA) can directly interfere with the core genes of circadian rhythm, thereby triggering an inflammatory cascade response and reproductive endocrine disorders (Yang et al., 2023; Zhao et al., 2021b). More notably, circadian rhythm disruption will further form a vicious cycle with oxidative stress and immunosuppression, thereby potentially amplifying the multi-organ damage effects of toxins (Deng et al., 2018; Ji et al., 2019).

This review will systematically explore the temporal regulation of toxin production and host infection by the fungal circadian rhythm, detailing the molecular pathways through which mycotoxins, such as DON and ZEA, disrupt the expression of core clock genes. Furthermore, it will investigate the complex feedback loop involving circadian rhythm disruption, oxidative stress, and immunosuppression, ultimately shedding light on the potential value and applications of targeting the circadian rhythm as a potential therapeutic strategy to mitigate mycotoxin-induced toxicity.

## 2 The regulation of toxin production and virulence by fungal circadian rhythm

To understand how mycotoxins disrupt the circadian rhythm, it is first essential to understand the molecular fundamentals of the circadian rhythm. Circadian rhythm constitutes an endogenous timing system that enables organisms to align physiological processes with daily light-dark cycles, maintaining a consistent 24-h periodicity (Lowrey and Takahashi, 2004; Preußner and Heyd, 2016; Yan et al., 2008). The circadian rhythm system operates through a molecular clock composed of interconnected transcriptional and translational feedback mechanisms (Asher and Sassone-Corsi, 2015; Dong et al., 2019). During the light phase, circadian locomotor output cycles kaput (*CLOCK*) and brain and muscle aryl hydrocarbon receptor nuclear translocator-like 1 (*BMAL1*) transcription factors form a heterodimer that binds to E-box elements, initiating the transcription of period (*PER1-3*) and cryptochrome (*CRY1/2*) genes (Mihut et al., 2025; Trujillo-Rangel et al., 2024). PER and CRY proteins accumulate in the cytoplasm and subsequently translocate to the nucleus, where they suppress *CLOCK-BMAL1*-driven transcription (Aiello et al., 2020; Touitou et al., 2017). Alterations in this regulatory system are associated with metabolic, inflammatory, oxidative stress, and immune pathologies (Curtis et al., 2014; Zhu et al., 2024).

The fungus circadian rhythm has been shown to regulate the daily timing of spore development and release (Bell-Pedersen et al., 1996; Hevia et al., 2016). For example, the *frequency* gene is considered a key component in regulating and maintaining the fungus circadian rhythm of *Neurospora crassa* (Bell-Pedersen et al., 1996). Current evidence indicates that fungal circadian control of mycotoxin-producing fungi has been demonstrated in a few species, such as *Fusarium oxysporum*. The endogenous fungus circadian rhythm clock of *Fusarium oxysporum*, a plant pathogenic fungus, modulates its virulence by regulating zinc stress responses and secondary metabolism (Lu et al., 2025). *F. oxysporum* exhibits daily fluctuations in host infestation, which are driven by its intrinsic fungus circadian rhythm (Lu et al., 2025). Specifically, the transcription factor *FoZafA* regulates adaptation to zinc-deficient environments, while the transcription factor *FoCzf1* governs fusaric acid biosynthesis (Lu et al., 2025). These findings indicate that fungus circadian rhythm in *F. oxysporum* orchestrates the temporal control of pathogenicity-related gene expression (Lu et al., 2025). This work underscores the endogenous fungus circadian rhythm plays a critical role in regulating both the pathogenic development and host penetration capacity of *F. oxysporum*. Moreover, if biosynthetic genes are downstream of circadian regulators, toxin levels can oscillate in a circadian manner (Schmidt-Heydt et al., 2010). The expression of the OTA polyketide synthase gene, a central component in the OTA biosynthetic pathway, exhibits rhythmic variation under light-dark cycles (Schmidt-Heydt et al., 2010). Furthermore, OTA production fluctuates depending on whether the fungus is maintained under continuous darkness or continuous illumination (Schmidt-Heydt et al., 2010). However, the generalizability of these mechanisms across the broad spectrum of mycotoxin-producing fungi requires further investigation.

## 3 The disruptive effects of mycotoxins on circadian rhythm gene expression

Fungi utilize their circadian rhythm to optimize host invasion and toxin production. Once inside the host, however, mycotoxins can directly interfere with the host circadian rhythm clock, which regulates physiological defense mechanisms. Growing evidence indicates that mycotoxins disrupt host circadian rhythm function, potentially accelerating disease progression (Yang et al., 2023; Zhao et al., 2021b). For example, key circadian rhythm genes were significantly downregulated upon DON exposure, suggesting disruption of host circadian rhythm (Yang et al., 2023). In Hepa 1-6 cells (mouse), RT-qPCR analysis revealed that *CLOCK*, *REV-ERBα*, *REV-ERBβ*, *CRY1*, and *CRY2* transcripts were all downregulated following DON exposure (Yang et al., 2023). Under DON exposure in this *in vitro* model, *BMAL1* levels were significantly reduced across examined groups, with the most substantial decrease observed in the *BMAL1* ^shRNA^ + DON group (Yang et al., 2023). Further *in vivo* results in mice showed that *BMAL1* upregulation reduced inflammatory mediators, whereas DON exposure significantly elevated serum IL-6, IL-1β, and TNF-α levels (Yang et al., 2023). The underlying mechanism involves DON-induced pro-inflammatory cytokines such as IL-6, IL-1β, and TNF-α, which ultimately suppress the transcription of core clock genes. These findings identify *BMAL1* as a central circadian rhythm regulator capable of suppressing inflammation triggered by acute DON exposure, suggesting its potential as a molecular target for mitigating DON-related toxicity in future studies. Given the structural similarity between DON and T-2 toxin (type B and A trichothecenes, respectively), it is plausible that T-2 toxin may also disrupt host circadian rhythm and immune function, a hypothesis warranting further investigation.

Similarly, ZEA exposure significantly disrupted host circadian rhythm gene expression in TM3 and primary Leydig cells. ZEA reduced mRNA levels of *BMAL1*, *DBP*, *PER2*, and *NR1D1* in TM3 cells (Zhao et al., 2021b). In Leydig cells, *BMAL1* and *DBP* expression levels were markedly reduced following ZEA exposure (Zhao et al., 2021b). Although *PER2* and *NR1D1* expression was not consistently decreased at all time points, Cosinor analysis indicated loss of rhythmicity under ZEA treatment. Additionally, testosterone secretion was significantly diminished after 24 or 48 h of exposure to ZEA (Zhao et al., 2021b). The host circadian rhythm and steroidogenic effects of ZEA were further validated in mouse testes, confirming similar alterations observed in primary Leydig cells and TM3 cells (Zhao et al., 2021b). These findings suggest that ZEA appears to interfere with testosterone production by altering the host circadian rhythm in Leydig cells. However, the underlying molecular pathways remain undefined. The precise mechanism by which ZEA alters clock gene transcription, and whether it is mediated by ROS, cytokines, or receptor signaling, remains to be fully elucidated. Future work may involve suppressing key circadian rhythm regulators to assess reproductive functional changes under toxin exposure.

## 4 Oxidative stress–immunosuppression driven by circadian rhythm disruption

The disruption of the circadian rhythm by mycotoxins is not an isolated effect, it initiates a cascade of detrimental downstream consequences, prominently including dysregulation of oxidative stress and immune function (Liu et al., 2021; Xie et al., 2020). Although increasing attention has been paid to circadian rhythm disruption induced by oxidative stress, the effects of oxidative stress on circadian rhythm gene expression and the underlying mechanisms remain poorly understood (Chhunchha et al., 2020; Early et al., 2018). Elevated ROS levels induced oscillatory suppression of *CLOCK1*, *BMAL1*, *PER3*, and *PER1* transcript abundance (Zhang et al., 2025). Disruption of *zeste homologue 2* (*EZH2*) led to a periodic decrease in the expression levels of core circadian rhythm genes. JTK-Cycle analysis confirmed that *EZH2* deficiency markedly reduced the amplitude of these genes (Zhang et al., 2025). *EZH2* overexpression markedly enhanced the circadian rhythm expression of *BMAL1* and *PER2*, while oxidative stress counteracted this effect by reducing the period length, elevating the amplitude, and inducing phase alterations (Zhang et al., 2025). These results strongly support the role of *EZH2* as a central modulator of ROS-dependent circadian rhythm control. Furthermore, *EZH2* promoted *CLOCK1*-*BMAL1*-driven *PER1* promoter activity in a dose-dependent manner, whereas oxidative stress inhibited this effect (Zhang et al., 2025). Therefore, oxidative stress alters circadian rhythm via ROS, which modulate the interaction between enhancer of *EZH2* and the *CLOCK*-*BMAL1* complex. In contrast, another study reported that oxidative stress enhanced the protein levels of circadian rhythm regulators CLOCK, BMAL1, PER1/2, and CRY1/2 in NIH3T3 cells (Ji et al., 2019). It also altered the oscillatory activity of BMAL1-luciferase through modulation of RORα, REV-ERBα, and REV-ERBβ (Ji et al., 2019). Subsequent analyses indicated that oxidative stress influences circadian rhythm timing via the signal transducer and activator of transcription 3 (STAT3)-REV-ERBα/β-peroxiredoxin-2 signaling axis (Ji et al., 2019). The apparent discrepancy between transcriptional repression and elevated protein levels may stem from multi-layered post-transcriptional regulation. This reason could be explained by biological mechanisms such as post-transcriptional compensation, where the cell may enhance the translation or stability of clock proteins. Additionally, the specific outcome may depend on critical experimental variables, including different oxidative stress levels/timing, with acute versus chronic exposure potentially triggering distinct cellular responses. Therefore, these opposing findings likely represent different facets of the circadian clock’s adaptive and maladaptive responses to oxidative stress.

**FIGURE 1:**
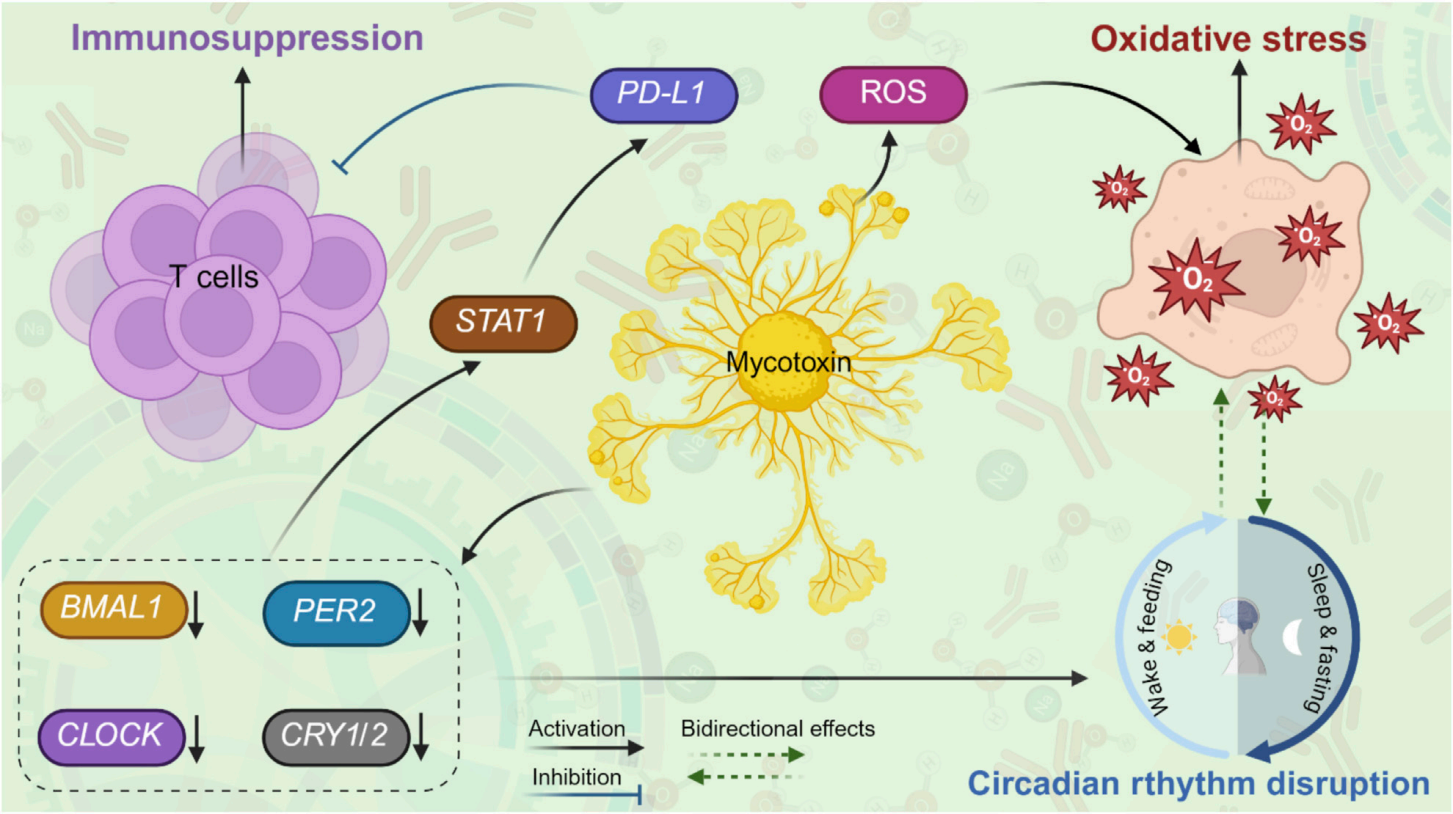
“A proposed model” of interactions between mycotoxins, circadian rhythm disruption, oxidative stress, and immunosuppression. Upon exposure to mycotoxins, mycotoxins downregulate the levels of core clock genes (*BMAL1*, *CLOCK*, *PER2*, *CRY1/2*), leading to circadian rhythm disruption and further exacerbating oxidative stress. In addition, mycotoxins induce ROS, leading to oxidative stress and further disrupting circadian rhythm. Mycotoxin-induced circadian rhythm disruption may promote immunosuppression through activating STAT1 signaling and upregulating *PD-L1*, subsequently suppressing T-cell activity.

This circadian rhythm disruption, in turn, significantly contributes to immunosuppression. The circadian rhythm regulator *BMAL1* plays a crucial role in suppressing the expression of *programmed cell death ligand 1* (*PD*-*L1*) in activated macrophages and monocytes (Deng et al., 2018). When *BMAL1* is lost, levels of *pyruvate kinase M2* (*PKM2*) rise, leading to increased lactate production, which promotes the induction of *PD*-*L1* through a *STAT1*-dependent mechanism. By restraining *PD*-*L1* expression, *BMAL1* helps preserve T cell functionality (Deng et al., 2018). These findings highlight the relationship between circadian rhythm disturbances and immunosuppression. Notably, disruptions in circadian rhythm also induce oxidative stress, exacerbating immunosuppression. Specifically, the deletion of *BMAL1*, which disrupts circadian rhythm, resulted in elevated levels of ROS and an upregulation of *PD*-*L1* (Fortin et al., 2024). Furthermore, the impairment of circadian rhythm was associated with an expansion of neutrophil and monocyte populations, while simultaneously decreasing the abundance of CD8^+^ T cells in both tumor-free and tumor-bearing tissues (Fortin et al., 2024). These observations confirm that circadian rhythm disruption plays a pivotal role in modulating immunosuppression. The circadian rhythm disruption induces oxidative stress and elevates ROS levels, which promotes PD-L1 upregulation and leads to immunosuppression. Additionally, the findings suggest a bidirectional relationship between oxidative stress and circadian rhythm, wherein each can exacerbate the other. Figure 1 illustrates how mycotoxins exposure triggers circadian rhythm disruption, oxidative stress, and immunosuppression via intricate molecular signaling pathways.

## 5 Concluding remarks and perspectives

Mycotoxins such as DON and ZEA demonstrably disrupt the expression and function of core circadian rhythm clock genes (such as *BMAL1* and *CLOCK*), leading to molecular and functional circadian rhythm disturbances. However, current knowledge remains limited regarding the specific mechanistic pathways by which circadian rhythm disruption contributes to the spectrum of mycotoxin-induced toxicities, including metabolic dysregulation, inflammation, oxidative stress, and immune dysfunction. The precise molecular pathways that link circadian rhythm disruption to the toxicity caused by mycotoxins (metabolic disorders, inflammation, oxidative stress, and immune dysfunction) remain to be explored. The dose-response and time-process relationship of various mycotoxins to circadian rhythm disruption remains unclear. It is crucial to determine whether these effects are transient and reversible or lead to long-term maladjustment of the circadian rhythm system, even after exposure ceases. The complex bidirectional relationship between circadian rhythm disruption and oxidative stress, positioning both as central mediators of mycotoxin-induced immunotoxicity. Future research could employ multi-omics methods and conditional knockout models to dissect this crosstalk and determine which node (for example, *EZH2* and *BMAL1*-*PKM2*-*PD-L1*) is the most effective intervention lever. Given the structural similarity between T-2 toxin and DON, its potential to disrupt the circadian rhythm and subsequent immune regulation deserves immediate investigation. Further research is urgently needed to elucidate the precise role of the circadian rhythm and to evaluate its potential as a novel intervention target for mitigating the multifaceted toxicity of mycotoxins.

## References

[B1] Aiello I. Mul Fedele M. L. Román F. Marpegan L. Caldart C. Chiesa J. J. (2020). Circadian disruption promotes tumor-immune microenvironment remodeling favoring tumor cell proliferation. Sci. Adv. 6 (42), eaaz4530. 10.1126/sciadv.aaz4530 33055171 PMC7556830

[B2] Asher G. Sassone-Corsi P. (2015). Time for food: the intimate interplay between nutrition, metabolism, and the circadian clock. Cell 161 (1), 84–92. 10.1016/j.cell.2015.03.015 25815987

[B3] Bell-Pedersen D. Garceau N. Loros J. J. (1996). Circadian rhythms in fungi. J. Genet. 75 (3), 387–401. 10.1007/BF02966317

[B4] Chhunchha B. Kubo E. Singh D. P. (2020). Clock protein Bmal1 and Nrf2 cooperatively control aging or oxidative response and redox homeostasis by regulating rhythmic expression of Prdx6. Cells 9 (8), 1861. 10.3390/cells9081861 32784474 PMC7463585

[B5] Curtis A. M. Bellet M. M. Sassone-Corsi P. O'Neill L. A. (2014). Circadian clock proteins and immunity. Immunity 40 (2), 178–186. 10.1016/j.immuni.2014.02.002 24560196

[B6] Darbuka E. Gürkaşlar C. Yaman I. (2021). Ochratoxin A induces ERK1/2 phosphorylation-dependent apoptosis through NF-κB/ERK axis in human proximal tubule HK-2 cell line. Toxicon 199, 79–86. 10.1016/j.toxicon.2021.06.005 34116085

[B7] Deng W. Zhu S. Zeng L. Liu J. Kang R. Yang M. (2018). The circadian clock controls immune checkpoint pathway in sepsis. Cell Rep. 24 (2), 366–378. 10.1016/j.celrep.2018.06.026 29996098 PMC6094382

[B8] Dong Z. Zhang G. Qu M. Gimple R. C. Wu Q. Qiu Z. (2019). Targeting glioblastoma stem cells through disruption of the circadian clock. Cancer Discov. 9 (11), 1556–1573. 10.1158/2159-8290.Cd-19-0215 31455674 PMC6983300

[B9] Early J. O. Menon D. Wyse C. A. Cervantes-Silva M. P. Zaslona Z. Carroll R. G. (2018). Circadian clock protein BMAL1 regulates IL-1β in macrophages via NRF2. Proc. Natl. Acad. Sci. U. S. A. 115 (36), E8460–E8468. 10.1073/pnas.1800431115 30127006 PMC6130388

[B10] Fortin B. M. Pfeiffer S. M. Insua-Rodríguez J. Alshetaiwi H. Moshensky A. Song W. A. (2024). Circadian control of tumor immunosuppression affects efficacy of immune checkpoint blockade. Nat. Immunol. 25 (7), 1257–1269. 10.1038/s41590-024-01859-0 38806707 PMC11374317

[B11] Hevia M. A. Canessa P. Larrondo L. F. (2016). Circadian clocks and the regulation of virulence in fungi: getting up to speed. Semin. Cell Dev. Biol. 57, 147–155. 10.1016/j.semcdb.2016.03.021 27039027

[B12] Ji G. Lv K. Chen H. Wang Y. Zhang Y. Li Y. (2019). Hydrogen peroxide modulates clock gene expression via PRX2-STAT3-REV-ERBα/β pathway. Free Radic. Biol. Med. 145, 312–320. 10.1016/j.freeradbiomed.2019.09.036 31585206

[B13] Kościelecka K. Kuć A. Kubik-Machura D. Męcik-Kronenberg T. Włodarek J. Radko L. (2023). Endocrine effect of some mycotoxins on humans: a clinical review of the ways to mitigate the action of mycotoxins. Toxins 15 (9), 515. 10.3390/toxins15090515 37755941 PMC10535190

[B14] Le G. Yuan X. Hou L. Ge L. Liu S. Muhmood A. (2020). Ochratoxin A induces glomerular injury through activating the ERK/NF-κB signaling pathway. Food Chem. Toxicol. 143, 111516. 10.1016/j.fct.2020.111516 32615238

[B15] Li P. Li K. Zou C. Tong C. Sun L. Cao Z. (2020). Selenium yeast alleviates ochratoxin A-induced hepatotoxicity via modulation of the PI3K/AKT and Nrf2/Keap1 signaling pathways in chickens. Toxins 12 (3), 143. 10.3390/toxins12030143 32106596 PMC7150738

[B16] Li E. Horn N. Ajuwon K. M. (2021). Mechanisms of deoxynivalenol-induced endocytosis and degradation of tight junction proteins in jejunal IPEC-J2 cells involve selective activation of the MAPK pathways. Arch. Toxicol. 95 (6), 2065–2079. 10.1007/s00204-021-03044-w 33847777

[B17] Li S. Liu R. Xia S. Wei G. Ishfaq M. Zhang Y. (2022). Protective role of curcumin on aflatoxin B1-induced TLR4/RIPK pathway mediated-necroptosis and inflammation in chicken liver. Ecotoxicol. Environ. Saf. 233, 113319. 10.1016/j.ecoenv.2022.113319 35189522

[B18] Liu X. Xiao W. Jiang Y. Zou L. Chen F. (2021). Bmal1 regulates the redox rhythm of hSPB1, and homooxidized HSPB1 attenuates the oxidative stress injury of cardiomyocytes. Oxid. Med. Cell. Longev. 2021, 5542815. 10.1155/2021/5542815 34239687 PMC8238613

[B19] Lowrey P. L. Takahashi J. S. (2004). Mammalian circadian biology: elucidating genome-wide levels of temporal organization. Annu. Rev. Genomics Hum. Genet. 5, 407–441. 10.1146/annurev.genom.5.061903.175925 15485355 PMC3770722

[B20] Lu Q. Yu M. Sun X. Zhou X. Zhang R. Zhang Y. (2025). Circadian clock is critical for fungal pathogenesis by regulating zinc starvation response and secondary metabolism. Sci. Adv. 11 (13), eads1341. 10.1126/sciadv.ads1341 40153515 PMC11952111

[B21] Mihut A. O'Neill J. S. Partch C. L. Crosby P. (2025). PERspectives on circadian cell biology. Philos. Trans. R. Soc. Lond. B Biol. Sci. 380 (1918), 20230483. 10.1098/rstb.2023.0483 39842483 PMC11753889

[B22] Moldal T. Bernhoft A. Rosenlund G. Kaldhusdal M. Koppang E. O. (2018). Dietary deoxynivalenol (DON) may impair the epithelial barrier and modulate the cytokine signaling in the intestine of Atlantic salmon (*Salmo salar*). Toxins 10 (9), 376. 10.3390/toxins10090376 30223534 PMC6162859

[B23] Preußner M. Heyd F. (2016). Post-transcriptional control of the mammalian circadian clock: implications for health and disease. Pflügers Arch. 468 (6), 983–991. 10.1007/s00424-016-1820-y 27108448 PMC4893061

[B24] Ruan D. Wang W. C. Lin C. X. Fouad A. M. Chen W. Xia W. G. (2019). Effects of curcumin on performance, antioxidation, intestinal barrier and mitochondrial function in ducks fed corn contaminated with ochratoxin A. Animal 13 (1), 42–52. 10.1017/s1751731118000678 29644962

[B25] Schmidt-Heydt M. Bode H. Raupp F. Geisen R. (2010). Influence of light on ochratoxin biosynthesis by Penicillium. Mycotoxin Res. 26 (1), 1–8. 10.1007/s12550-009-0034-y 23605235

[B26] Shekhar R. Raghavendra V. B. Rachitha P. (2025). A comprehensive review of mycotoxins, their toxicity, and innovative detoxification methods. Toxicol. Rep. 14, 101952. 10.1016/j.toxrep.2025.101952 40162074 PMC11954124

[B27] Sun Y. Huang K. Long M. Yang S. Zhang Y. (2022). An update on immunotoxicity and mechanisms of action of six environmental mycotoxins. Food Chem. Toxicol. 163, 112895. 10.1016/j.fct.2022.112895 35219766

[B28] Tian F. Woo S. Y. Lee S. Y. Park S. B. Im J. H. Chun H. S. (2022). Mycotoxins in soybean-based foods fermented with filamentous fungi: occurrence and preventive strategies. Compr. Rev. Food Sci. Food Saf. 21 (6), 5131–5152. 10.1111/1541-4337.13032 36084140

[B29] Touitou Y. Reinberg A. Touitou D. (2017). Association between light at night, melatonin secretion, sleep deprivation, and the internal clock: health impacts and mechanisms of circadian disruption. Life Sci. 173, 94–106. 10.1016/j.lfs.2017.02.008 28214594

[B30] Trujillo-Rangel W. Á. Acuña-Vaca S. Padilla-Ponce D. J. García-Mercado F. G. Torres-Mendoza B. M. Pacheco-Moises F. P. (2024). Modulation of the circadian rhythm and oxidative stress as molecular targets to improve vascular dementia: a pharmacological perspective. Int. J. Mol. Sci. 25 (8), 4401. 10.3390/ijms25084401 38673986 PMC11050388

[B31] Wang W. Zhai S. Xia Y. Wang H. Ruan D. Zhou T. (2019). Ochratoxin A induces liver inflammation: involvement of intestinal microbiota. Microbiome 7 (1), 151. 10.1186/s40168-019-0761-z 31779704 PMC6883682

[B32] Wang P. Huang L. Yang W. Liu Q. Li F. Wang C. (2021). Deoxynivalenol induces inflammation in the small intestine of weaned rabbits by activating mitogen-activated protein kinase signaling. Front. Vet. Sci. 8, 632599. 10.3389/fvets.2021.632599 33604367 PMC7884333

[B33] Wang J. Zhang F. Yao T. Li Y. Wei N. (2022). Risk assessment of mycotoxins, the identification and environmental influence on toxin-producing ability of alternaria alternate in the main Tibetan Plateau triticeae crops. Front. Microbiol. 13, 1115592. 10.3389/fmicb.2022.1115592 36824588 PMC9942522

[B34] Xie M. Tang Q. Nie J. Zhang C. Zhou X. Yu S. (2020). BMAL1-downregulation aggravates porphyromonas gingivalis-induced atherosclerosis by encouraging oxidative stress. Circ. Res. 126 (6), e15–e29. 10.1161/circresaha.119.315502 32078488

[B35] Yan J. Wang H. Liu Y. Shao C. (2008). Analysis of gene regulatory networks in the mammalian circadian rhythm. PLoS Comput. Biol. 4 (10), e1000193. 10.1371/journal.pcbi.1000193 18846204 PMC2543109

[B36] Yang L. N. Xu S. Tang M. Zhou X. Liao Y. Nüssler A. K. (2023). The circadian rhythm gene Bmal1 ameliorates acute deoxynivalenol-induced liver damage. Arch. Toxicol. 97 (3), 787–804. 10.1007/s00204-022-03431-x 36602574

[B37] Zhang H.-y. Li K.-y. Wang Y.-l. Wei C. J. Gao Y. X. Ren-Zhou (2025). ROS regulates circadian rhythms by modulating Ezh2 interactions with clock proteins. Redox Biol. 81, 103526. 10.1016/j.redox.2025.103526 39952198 PMC11875201

[B38] Zhao L. Deng J. Xu Z. J. Zhang W. P. Khalil M. M. Karrow N. A. (2021a). Mitigation of aflatoxin B(1) hepatoxicity by dietary hedyotis diffusa is associated with activation of NRF2/ARE signaling in chicks. Antioxidants (Basel) 10 (6), 878. 10.3390/antiox10060878 34070870 PMC8229166

[B39] Zhao L. Xiao Y. Li C. Zhang J. Zhang Y. Wu M. (2021b). Zearalenone perturbs the circadian clock and inhibits testosterone synthesis in mouse Leydig cells. J. Toxicol. Environ. Health A 84 (3), 112–124. 10.1080/15287394.2020.1841699 33148124

[B40] Zhu X. Maier G. Panda S. (2024). Learning from circadian rhythm to transform cancer prevention, prognosis, and survivorship care. Trends Cancer 10 (3), 196–207. 10.1016/j.trecan.2023.11.002 38001006 PMC10939944

